# Effect of increasing levels of wasted date palm in concentrate diet on reproductive performance of Ouled Djellal breeding rams during flushing period

**DOI:** 10.14202/vetworld.2018.712-719

**Published:** 2018-05-28

**Authors:** A. Allaoui, B. Safsaf, M. Tlidjane, I. Djaalab, H. Djaalab Mansour

**Affiliations:** 1 Department of Veterinary Sciences, Veterinary and Agricultural Sciences Institute, Laboratory ESPA, Hadj Lakhdar Batna-1 University, Batna - 05000, Algeria; 2 Department of Animal Productions, Veterinary Sciences Institute, Laboratory GSPA, Mentouri University, Constantine -25000, Algeria

**Keywords:** body weight, flushing period, rams, semen, wasted date

## Abstract

**Aim:**

The aim of the study was to assess the effect of two levels of wasted date (WD) by replacing commercial concentrate on the reproductive performance of Ouled Djellal (OD) rams.

**Materials and Methods:**

Eighteen mature (2-year-old) OD rams were equally allocated to three groups and fed during 11 weeks with one of three different experimental diets, that contained 0% (0 WD), 50% (50 WD), or 75% (75 WD) of WDs in concentrate diet. Live body weight (LBW), body condition scoring (BCS), scrotal circumference (SC), testicular weight (TW), sperm production and quality, plasma testosterone concentration (T), and sexual behavior (reaction time and number of mounts with ejaculation) were regularly recorded from every ram.

**Results:**

LBW, SC, and TW changed significantly among diet groups and during the experimental period (p<0.001), the highest averages were recorded in (0 WD) group. LBW, BCS, SC, TW, semen volume, and percentage of the positive hypo-osmotic swelling test were (p<0.001) positively influenced by flushing period. Nevertheless, sperm concentration showed a significant (p<0.001) decrease at day 30, followed by a return to the initial values afterward. There were no differences (p>0.05) between diet groups for plasma testosterone concentration and semen attributes, except that (50 WD) group expressed the lowest overall value of semen concentration. Furthermore, neither time nor diet affected (p>0.05) sperm motility and reproductive behavior parameters.

**Conclusions:**

It is possible to introduce WD as unconventional local feeding resources in flushing diet of breeding rams without disturbing their reproductive performance.

## Introduction

In Algeria, the sheep occupy an important socioeconomic position that can be appreciated through the high effective population which exceeds 26 million [1] and the diversity of races [2,3].

The local sheep breeds are mostly reared in an agro-pastoral extensive system under an arid to semi-arid climate [4]. In this system, grazing pastures play a paramount role in livestock feed. However, overgrazing and climatic variations cause instability of the quantitative and qualitative nutritive value of the forage, which remains mediocre and below the needs of animals [5]. In addition, the potential for using conventional cereals for sheep feed is limited, due to their low yields, irregular production, and high cost [6,7]. This often leads to deficient and unbalanced diets impacting negatively on the reproductive performance of both male and female [8].

Ovine artificial insemination is insufficiently developed in Algeria [9]. Farmers usually use the natural mating method, with two seasons on the program: Spring (March-May) and autumn (September-November) [10]. Breeding rams are crucial in this management system; their individual impact on multiple pregnancies is major. Flushing, i.e., increased energy and protein availability, in the ration, during 2 months before the fight, has a determining effect on the fertility of rams, by conditioning their quality of semen, sexual behavior, and body condition at mating [11]. However, the seasonal availability of pasture and the sustained rise in the price of primary animal feed ingredients leave farmers with little choice for improving the diet of spawners.

A wide range of alternative feed sources has proved efficient in maintaining or even improving, the reproductive performance of the flock [12]. With a total production of 990.000 tonnes of date [13], Algeria is the fourth important countries in date world production [14]. Date residues may constitute a good alternative to other cereal products as they contain carbohydrates and minerals. In addition, they present a high digestibility coefficient except for proteins [15]. These by-products provide one solution to optimize our livestock productive and reproductive performance at low cost. The specific effects of their incorporation in diets were studied on growing sheep [16,17] and reproductive ewes [18,19]. However, only a few results on the response of breeding rams are presented.

In the light of these observations, this study was planned to compare the effect of incorporation of wasted date (WD) palm in concentrate diet at a ratio of 0% (WD), 50% (WD), or 75% (WD), on the performance of reproduction of ram, during the flushing period, to determine which diet has the best influence on sperm production and body measurements.

## Materials and Methods

### Ethical approval

The ethical considerations in accordance to the Institute Animal Ethics Committee related to animal handling were observed to ensure no pain to animal during the different samplings.

### Site, animals, and experimental diets

The study was conducted on the state experimental farm of Bouaoun Rabah, which is located in El-khroub (Constantine, Eastern Algeria, with a Mediterranean climate type continental semi-arid) at latitude 36°15′47″North and longitude 6°41′36″East at a mean altitude of 630 meters above sea level. 18 healthy sexually mature (2-year-old) Ouled Djellal (OD) rams, with a mean (± standard error of mean [S.E.M.]), live body weight (LBW) of 63.66±0.42 kg and scrotal circumference (SC) of 31.32±0.11 cm, were selected with proven libido from the main flock in the experimental farm. Rams, previously raised on natural pastures, were separated from ewes since June 7, and reared in individually 2.5 m×1.5 m pens in a well-ventilated covered building. They were randomly allocated equally to three groups, for each group the diet was changed gradually during the first 2 weeks, the data collection started after this adaptation period and lasted 11 weeks. Table-1 shows the ingredients, the quantities and the chemical composition of the diet fed to the rams. Group (0 WD) was kept as a control and was fed the flushing diet used usually in the farm. The second (50 WD) and third (75 WD) groups were fed an experimental diet in which 50% and 75%, respectively, of a commercial concentrate (80% crushed barley, 10% wheat bran, 7% soybean oilcake, and 3% Mineral-vitamin mixture) were replaced with discarded dates (low-quality date fruits with kernels and unfit for human consumption). Feeding was designed to supply approximately 1.2 times the maintenance requirements of animals [20]. Concentrate mixture was distributed in two equal portions at 9:00 and 16:00 h daily before hay feeding. Freshwater and a salt block were freely available.

**Table 1:** Ingredients and chemical composition of experimental diets.

Ingredients and chemical composition	Diets		

	0 WD	50 WD	75 WD
Ingredients			
Hay (kg/head/day)	1.5	1.5	1.5
Concentrated mixture (kg/head/day)	1	1	1
Wasted date (% of CM)	0	50	75
Commercial concentrate (% of CM) concentrate	100	50	25
Chemical composition			
DM (g/kg)	930.6	912.3	903.2
Organic matter (% of DM)	97.30	96.52	96.13
Crude protein (% of DM)	11.80	10.29	9.54
Neutral detergent fiber (% of DM)	56.08	49.53	46.25
Acid detergent fiber (% of DM)	27.06	25.19	24.25
Cellulose (% of DM)	17.57	14.28	12.63
Hemicelluloses (% of DM)	29.02	24.34	22.00
Lignin (% of DM)	9.49	10.91	11.62

CM=Concentrated mixture, DM=Dry matter, WD=Wasted date

### Physical measurements and blood samples

LBW, body condition scoring (BCS), SC, testicular weight (TW), and plasma testosterone concentration (T) were recorded from every ram on days 1, 15, 29, 43, 57, and 71. All these measurements were done in the morning before feeding. LBW was performed using an electronic beast scale, then the body weight gain percentage (BWG %) [21] and the average daily gain (ADG g/day) were calculated. BCS was judged by palpation of the sacrolumbar area and noted from 1 to 5 as previously described by Maurya *et al*. [22]. SC was measured with a metrical tape at the widest scrotal diameter. TW was measured volumetrically, using the Archimedes principles of water displacement. The ram was in a standing position, the whole scrotal sac was immersed in beaker filled with water of 2 L capacity, the amount of water displaced corresponds to the testicular volume, knowing that the mass of 1 mL (1 cm^3^) equals 1 g; therefore, the TW can be deduced from the scrotal sac volume [23]. Blood samples (10 ml) were collected from the jugular vein using heparinized evacuated tubes and immediately centrifuged at 3500× *g* for 20 min. Plasma was prepared and stored at −20°C until assay. T was determined using a testosterone Electrochemiluminescence Kit (Roche Diagnostics, Germany, Measuring range: 0.025-15.0 ng/mL).

### Semen collection and evaluation

Semen samples were collected, at 06:30 h, on days 2, 30, 58, and 72 of the experimental period. Semen was harvested using electroejaculation [24]. The automatic mode (Electro Ejaculator e320 minitüb Germany) suitable for small ruminants was used by increasing the power output of the rectal probe linearly from 0.5 to 7 V at 2 s intervals. Immediately after ejaculation, volume (measured directly from the graduated collecting tube), appearance, and consistency (scored on a scale of 0-5) of semen were estimated [25]. Following this macroscopic evaluation semen samples were diluted 1:1 v/v with a pre-warmed (37°C) commercial extender (Andromed), kept in a hot/cold thermoelectric cooler (12/230 V) at 37°C and transported to the laboratory of the biotechnology research center, for evaluation within 35 min after collection. At the laboratory, the ejaculates were transferred to a water bath (37°C) and were evaluated for mass motility (score from 0 [immotile] to 5 [rapid wave motion, eddies present]) [26], concentration (using a photometer previously calibrated with a hemocytometer), total motility (TM %) (using a computer-assisted semen analyzer [CASA] [Sperm class analyzer, SCA Microptic, S.L., Version 3.2.0, Barcelona, Spain]) [27], and membrane integrity of sperm (using the hypo-osmotic swelling test [HOST]) [28].

### Sexual behavior

Reaction time (time from entry into the mating pens to the first mount with ejaculation) and number of mounts with ejaculation were recorded on days: 7, 35, and 77 of the experimental period. The test was conducted by exposing each ram to four estrous ewes for 15 min [29]. The induction of estrus in ewes was done by hormonal treatments, as previously described by Allaoui *et al*. [9].

### Statistical analysis

The effect of fixed factors, diet (0 WD, 50 WD, and 75 WD) and time (day) of the trial and also their interaction, on the various studied parameters, were analyzed using a two-way analysis of variance. Tukey’s multiple comparisons test was conducted to test significance between means of the different subgroups. Differences were considered significant when p<0.05. All mean values are expressed as the mean ± S.E.M. Correlation coefficients (Pearson correlations) between various physical and seminal parameters were calculated. All analyses were performed using GraphPad Prism 7.00 software.

## Results

### LBW, BCS, and scrotal measurements

The effect of diet and day on LBW, ADG, and BCS and scrotal measurements was described in Table-2. Furthermore, correlation coefficients between physical measurements, plasma testosterone concentration, and experimental day were described in Table-3. For all groups LBW increased significantly (p<0.001) during the study period, mean values were higher (p<0.001) in 0 WD than 50 WD and 75WD with observed BWG% of 21.57±2.17%, 12.52±1.52%, and 14.59±1.64% for 0 WD, 50 WD, and 75 WD, respectively. ADG was significantly (p<0.001) affected by the experimental day, averages were significantly (p<0.05) higher in 0WD as compared with 50 WD. BCS was strongly correlated with LBW and experimental day (Table-3), it increased significantly (p<0.001) during the study period, mean values were higher (p<0.05) in 0 WD group. Overall, the effect of diet and the experimental day was significant (p<0.001) on SC and TW, values were higher (p<0.001) in 0 WD than 75 WD group but there was no difference (p>0.05) between 0 WD and 50 WD.

**Table-2:** Effects of diet and experimental day on LBW, BCS, and scrotal measurement of OD rams receiving 75%, 50%, or 0% of wasted date in the concentrate diet.

Factors	LBW (kg)	ADG (g/day)	BCS (1-5)	SC (cm)	TW (g)
Diet	***	*	*	***	***
75 WD	68.91±1.69	137.68±16.46	2.72±0.06	31.45±0.18	689.86±13.79
50 WD	68.22±0.63	110.83±12.57	2.73±0.03	32.14±0.27	781.53±21.77
0 WD	72.13±1.08	193.90±18.40	2.85±0.08	32.64±0.65	794.36±32.39
Day	***	***	***	***	***
1	63.71±0.27		2.38±0.00	31.87±0.16	691.39±13.35
15	66.31±0.67	173.15±37.28	2.58±0.02	31.47±0.42	678.89±35.10
29	69.23±1.30	195.26±52.04	2.78±0.07	30.99±0.13	699.17±37.53
43	71.93±1.64	180.00± 1;41.93	2.85±0.06	31.93±0.55	776.78±36.37
57	73.18±1.74	84.26±23.83	2.99±0.05	33.03±0.47	844.17±38.50
71	74.18±1.85	66.48±23.18	3.04±0.06	33.18±0.44	841.11±40.84
Diet x Day	NS	NS	NS	NS	NS

* p<0.05,

*** p<0.001. LBW=Live body weight, ADG=Average daily gain, BCS=Body condition scoring, SC=Scrotal circumference, TW=Testicular weight. NS=Non-significant, OD=Ouled Djellal, WD=Wasted date

**Table-3:** Correlation coefficients between physical measurements, plasma testosterone concentration, and day of trial in rams receiving 75%, 50%, or 0% of wasted date in the concentrate diet.

Diet	(75 WD)					(50 WD)					(0 WD)				
			
Para-meter	BSC (1-5)	LBW (Kg)	SC (cm)	TW (g)	T (ng/mL)	BSC (1–5)	LBW (Kg)	SC (cm)	TW (g)	T (ng/mL)	BSC (1–5)	LBW (Kg)	SC (cm)	TW (g)	T (ng/mL)
LBW	0.***					0.74***					0.90***				
SC	0.30 NS	0.48**				0.40*	0.35*				0.43**	0.50**			
TW	0.43**	0.58***	0.79***			0.46**	0.47**	0.***			0.66***	0.79***	0.79***		
T	0. 47***	0.57***	0.61***	0.64***		0.53***	0.76***	0.30 NS	0.27 NS		0.75***	0.83***	0.34*	0.57***	
Day	0.74***	0.63***	0.46**	0.57***	0.77***	0.81***	0.85***	0.39*	0.59***	0.71***	0.72***	0.86***	0.44**	0.69***	0.79***

* p<0.05,

** p<0.01,

*** p<0.001. BCS=Body condition scoring, LBW=Live body weight, SC=Scrotal circumference, TW=Testicular weight, T=Plasma testosterone concentration, NS=Non-significant

### Plasma testosterone concentration

**Figure-1:**
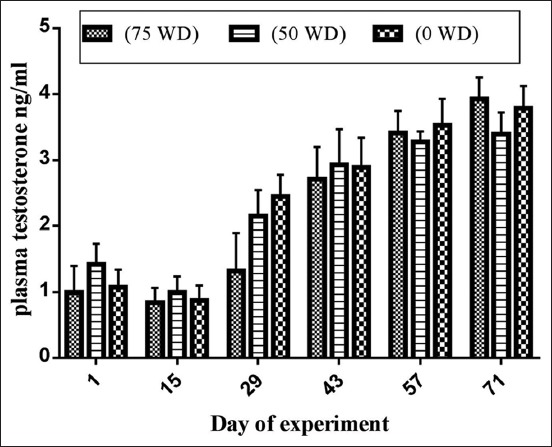
Mean±(standard error of mean) plasma testosterone concentrations on days 1, 15, 29, 43, 57, and 71 for Ouled Djellal rams receiving 75%, 50%, or 0% of wasted date in the concentrate diet.

The average values of testosterone levels are presented in Figure-1. Means were of 2.20±0.54 ng/mL, 2.36±0.41 ng/mL, and 2.43±0.50 ng/mL for 75 WD, 50 WD, and 0 WD group, respectively, with no significant (p>0.05) variance noted between groups. For all groups, values increased significantly (p<0.001) from day 1 to day 43 then they did not vary significantly (p<0.05) afterward.

### Seminal attributes and sexual behavior

The effects of diet and time on seminal parameters are detailed in Table-4. Overall means of reaction time and number of mount with service were, respectively, 27.96±2.5 s and 2.76±0.20. The data did not show significant differences in semen attributes between diet groups at any sampling time. However, the overall value of semen concentration (Table-4), for 50 WD appeared to be lower (p<0.05) compared to 0 WD group. Mean semen volume and percentage of sperm that was HOST test positive increased significantly (p<0.001) between day 2 and day 58 then remained invariable. Sperm concentration decreased significantly (p<0.001) from day 2 to day 30, afterward average values of both semen concentration and total production showed a rise (p<0.001) from day 30 to day 72. Sperm mass and TM, reaction time and number of mount with service were not affected (p>0.05) by either diet, time or diet x time interaction. The color of fresh semen was white creamy in all rams. Semen consistency was strongly correlated with semen concentration (r=0.91 p<0.001), and SC had the highest correlation coefficients with seminal parameters with correlation values ranging from 0.73 for total sperm production, 0.56 and 0.53 for sperm concentration and volume, respectively (p<0.001).

**Table-4:** Effects of diet and time on seminal parameters of OD rams receiving 75%, 50%, or 0% of wasted date in the concentrate diet.

Factors	Semen volume (ml)	Sperm concentration (×10^9^/ml)	Total sperm/ejaculate (×10^9^)	Sperm mass motility (0-5 scale)	Sperm TM (%)	HOST (%)
						
Diet	NS	*	NS	NS	NS	NS
75 WD	2.13±0.15	2.38±0.27	4.76±0.66	4.83±0.08	87.87±2.52	55.79±3.01
50 WD	2.08±0.22	1.81±0.21	4.02±0.74	4.63±0.13	87.36±1.42	52.75±2.96
0 WD	1.95±0.19	2.56±0.25	5.16±0.74	4.48±0.17	84.68±3.57	51.21±2.32

Day	***	***	***	NS	NS	***

2	1.61±0.14	2.71±0.28	4.39±0.29	4.58±0.08	85.70±1.41	45.89±1.64
30	1.81±0.12	1.37±0.07	2.41±0.17	4.56±0.15	84.85±1.88	46.94±1.79
58	2.46±0.07	2.28±0.28	5.78±0.59	4.83±0.10	89.12±0.48	60.06±1.58
72	2.33±0.08	2.64±0.32	6.01±0.41	4.61±0.15	86.88±0.60	60.11±1.47
Diet x Day	NS	NS	NS	NS	NS	NS

* p<0.05,

*** p<0.001. NS=Non-significant, HOST (%): Mean percentage of sperm that was HOST test positive. OD=Ouled Djellal, TN=Total motility, HOST=Hypo-osmotic swelling test

## Discussion

All components of the reproductive cycle are energy spending process. The expenditure of energy differs in its timing between the sexes, females invest most of their energy following fertilization, while the male needs more energy before fertilization, rams should thus be fed correctly for 2 months before mating [11,12].

As expected, for all groups, flush feeding influenced the nutritional condition of the animals positively consequently increasing the live weight and score body condition. Several studies [30-32] have demonstrated that feed supplementation in the form of flushing diet improves LBW, BCS, and reproductive performance of ewes. Similar observations have been reported in the mature Sardinian rams receiving a different level of concentrate (1.2 times or 1.5 times their maintenance requirements) [33]. However, Sakly *et al*. [34] noted that in Ile-de-France rams, short-term flushing with lupins does not cause any change in these parameters. In our study, LBW was too low at the beginning of the trial (Table-2), this can be justified by the fact that rams were involved in the previous mating season (spring season) which can lead to the weight loss [35]. After 2 months of supplementation, BCS increased significantly, reaching approximately the standard range (between 3 and 3.5) recommended by Maurya *et al*. [36] for breeding rams to exhibit better reproductive efficiency.

On the other hand, as the daily feeding level intake was similar for all groups, the gap observed between 0 WD group and the latter two groups in terms of live weight, BWG %, and ADG can probably be attributed to the differences in crude protein and fiber levels of the rations (Table-1). Rekik *et al*. [37] Boudechiche *et al*. [38], and Djaalab [39] found that compared to barley grains and hay, WDs were lower in crude protein and fiber, but higher in energy. Rekik *et al*. [37] added that partial or total substitution of barley by WDs resulted in a decrease of nitrogenous matter digestibility, associated with live weight decline in prolific D’Man ewes. To compensate for this shortage, some previous studies [40-42] suggested the addition of supplemental protein in the ration.

The general pattern of variation for scrotal measurements, semen volume, total sperm production, and percentage of sperm that was HOST test positive, showed a trend to increase during the experimental period, this is in agreement with several results [29,33,43-45] that confirm a strong relationship between nutrition and reproductive variables of ram. Martin *et al*. [11], Maurya *et al*. [36], and Blache *et al*. [46] also supported this hypothesis by explaining that in the long-term, a sustained increase in nutrition leads to an increase in both testis size and sperm production. On the contrary, Bester *et al*. [47] indicated that although higher energy concentration accelerates testicular development, it has no significant effect on semen quality and quantity of young Dorper rams.

For all experimental rams, semen attributes values were similar during all the trial period, except for concentration which appeared to be slightly lower in 50 WD group as compared to 0 WD and 75 WD. These differences may be related to the diet effect, or simply to a lower genetic potential of some rams of the group. In fact, for the OD ram, a previous study [40] suggested that inclusion of WD in the concentrate at level 75% has the best result on the digestibility of nutrients, in addition, for (0 WD) diet, the high energy level of the ration can result in better semen concentration. However, it is probably necessary to mention that the genetic potential and the adaptability of each animal play the most important role than other factors [11]. In this study also, exposure of the animals to combined stresses (change in the management system and nutritional stresses) during the adaptation period may explain the significant decreased in semen concentration observed at day 30 of the experiment (6 weeks later). These results are consistent with those obtained by Maurya *et al*. [48], who reported less concentration of sperm in Malpura rams maintained under conditions of nutritional or combined stresses.

It is well known that sperm motility is one of the important factors in semen evaluation and a convincing indicator of fertility in sheep [49]. The most of wave motion scoring recorded were between 4 and 5. Likewise, the proportions of actively motile spermatozoa evaluated using a CASA were mostly ≥70%, which means that ejaculates produced by rams were of high-quality [27,49] during all the study period.

Concerning testosterone production and sexual behavior, Martin *et al*. [11] noted that, except for extreme undernutrition, nutrition effects are not linked to major changes in these parameters. Thus, in our study, it seems clear that seasonal variations are the main factor influencing plasma testosterone concentration in the three groups. The greatest averages were recorded during the months (mid-July-August) that indicate the resumption of the natural breeding season in OD rams [23,50]. It should be noted that in our ovine breeds, the effect of season on testosterone levels is more marked than on live weight, testicular volume, and semen characteristics [23,50,51]. Furthermore, reaction time and number of mount with service remained invariable during all the experimental period and between dietary groups, similar observations were reported by Luna-Palomera *et al*. [52] in pubertal hair rams and Fernandez *et al*. [29] in mature Assaf rams receiving different levels of undegradable protein supply. Nevertheless, recent studies have demonstrated a significant effect of greater nutrition supplementation [22,45], or nutritional stress [48,53], on testosterone production, and sexual behavior expression in rams.

## Conclusion

In this study, we used the flushing diet used usually in the farm, the three groups are different only by the nature of the given energetic complements (WD vs. commercial concentrate), without adjusting the protein or caloric levels of the experimental diets. Our objective was to test if WDs (unconventional local feeding resources, of low cost) can economically and effectively substitute the conventional feedstuffs for breeding rams. As a result, we found that: (1) In general, and despite the variations between groups, flushing diets have a positive effect on growth performance, scrotal measurement, and seminal production of breeding rams. (2) It is possible to introduce WD in flushing diet of breeding rams without disturbing their productive and reproductive performance; therefore, it is possible to reduce the cost of feedstuffs and offer the possibility to the farmers to improve the diet of rams during the crucial times. (3) WD can be exploited as a substitute to the commercial concentrate at the level of 75% (the obtained results are slightly better than with a substitution at 50%) with a significant increase in LBW and seminal parameters of rams.

## Recommendations

To the best of our knowledge, this is the first study in which the effect of rations containing WD palm, on semen production, plasma testosterone concentration, and testicular size has been investigated in OD rams, that is why our future studies are going to be focused on using the same rations with adjusted protein level for a longer feeding period to see if there will be an improvement of the reproductive performance of males.

## Authors’ Contributions

MT, BS, AA, ID, and HDM designed the experiment protocol. AA carried out the experiment work at the experimental farm and the laboratory of biotechnology research center. ID performed the biochemical analysis of the wasted date, hay, and commercial concentrate used in the ration. AA, ID, BS, HDM, and MT were involved in data analysis and scientific discussion. All authors read and approved the final manuscript.
